# Improving health-care quality in resource-poor settings

**DOI:** 10.2471/BLT.16.170803

**Published:** 2016-11-30

**Authors:** Bejoy Nambiar, Dougal S Hargreaves, Chelsea Morroni, Michelle Heys, Sonya Crowe, Christina Pagel, Felicity Fitzgerald, Susana Frazao Pinheiro, Delan Devakumar, Sue Mann, Monica Lakhanpaul, Martin Marshall, Tim Colbourn

**Affiliations:** a Institute for Global Health, University College London, 30 Guilford Street, London, WC1N 1EH, England.; b Great Ormond Street Institute of Child Health, University College London, London, England.; c Institute for Women's Health, University College London, London, England.; d Clinical Operational Research Unit, University College London, London, England.; e School of Management, University College London, London, England.; f Centre for Infectious Disease Epidemiology, University College London, London, England.; g Department of Primary Care and Population Health, University College London, London, England.

Improvements in health-care quality can contribute to healthier populations. However, many global and national health strategies are not sufficiently considering the issues of measuring and improving health-care quality in low-resource settings.^1^ The barriers to delivering high-quality care are often similar across different health systems. However, the extent and mechanisms through which these barriers affect quality improvement interventions may be different in resource-poor settings.^2^ Investments in health systems strengthening without continuous quality improvement is thought to be a useless effort.^3^ Conversely, only focusing on quality improvement in a resource-poor context without engaging the broader health system for support is of limited value. Hence, both areas must be improved simultaneously.

Here, we call for renewed focus on quality improvement of health-system delivery by policy-makers, managers and health-care providers, working at all levels of health-care systems in resource-poor settings. To maximize the potential of quality improvements, we propose an approach focusing on five elements: (i) systems thinking; (ii) stakeholders’ participation; (iii) accountability; (iv) evidence-based interventions; and (v) innovative evaluation (Box 1). Some of the elements are well supported by peer-reviewed literature, while other elements are lacking good evidence. We base our ideas on our experience in diverse countries and settings. We hope that bringing all these elements together into a unified approach will stimulate debate, highlight important research gaps and support policy-makers, health-care providers and patient and community representatives working in this field.

Box 1 **Elements to consider when improving health-care quality in resource-poor settings**

**Systems thinking**

Health systems are dynamic complex adaptive systems, where all parts need to be considered. These parts are (i) the inter-relationships between the patient, clinical and nonclinical workers in the health system; (ii) the different levels of the health system ranging from the community to tertiary referral system; and (iii) the required human and material resources and training, supervision and management structures.

**Participatory approach**

Participatory, grounded and bottom-up approaches involving health-care professionals, patients and communities as well as researchers-in-residence are important to understand health systems. Participation also increases buy-in to quality improvement efforts and enables design and implementation of interventions that are effective in specific contexts, consider sociocultural beliefs and build accountability.

**Accountability**

The people involved in making health systems work must be accountable to the individuals and local communities the health system is serving. Data for decision-making is important as it can be used to encourage and track quality improvements and, when useful metrics are chosen, can also be a mechanism by which the health system can be held accountable.

**Evidence-based**

Evidence on what works to improve quality of care in low-resource settings is scarce. We propose an evidence-based approach that supports data harmonization while at the same time maintaining the highest standards of scientific and academic rigor.

**Innovative evaluation**

Both plausibility and probability evaluation designs should be used as part of a research strategy to rigorously determine whether quality improvement interventions can work and how, why and in what circumstances they work. Using a range of research strategies from theory-based evaluation to cluster randomized controlled trials is important.

The first element, systems thinking, views health-care systems in a holistic manner and is often described as operating at micro- (clinical team), meso- (health facilities) and macro-level (health-care system). Systems thinking offers a useful framework for addressing the interdependency of these different levels that influence health-care delivery and health outcomes.^4^ Interventions targeted to improve quality of care are unlikely to succeed or be sustained if designed without an adequate understanding of relevant contextual factors at these different levels.^5^ Researchers and implementers are now recognizing the importance of these factors and their dynamic interaction in delivering safer, more effective care.^6^ Failure to address these interdependencies may be particularly damaging in resource-poor settings, where constrained resources, lack of infrastructure and weak governance can exacerbate the difficulties of implementing an intervention. Examples range from unused expensive equipment due to lack of trained staff or electricity to operate it, to scarce health workers migrating to national and international organizations leading to understaffed primary, maternal and child care. Cultural and behavioural factors also affect these interdependencies and can contribute to problems during implementation. Interventions in resource-poor settings have to consider improvement across the different levels of the health system when trying to improve care at the clinical microsystems level.^7^

Second, local staff and communities should be engaged during the design phase of a systems improvement intervention. Key stakeholders, such as health-care providers and patients, bring experience and knowledge of local and national health-care systems. Furthermore, they understand the political, social, economic and cultural factors that influence health outcomes, use of health care and the implementation of interventions. In many high-income countries, stakeholders frequently participate in improvement of health-care practices.^8^ In resource-poor settings, participatory approaches may be useful to address potential conflicts between intervention philosophy and a wide range of cultural, social and economic factors, which could contribute to the success of the interventions.^5^^,^^8^ For example, participation approach could include tailoring quality improvement to specifically incorporate women- and family-friendly activities in contexts where a women’s role within the health-care system is relatively weak.

The organizational culture and readiness of a health system can influence stakeholder participation and engagement. A better understanding of the culture and practice for improvement is required for stakeholder engagement and participation, and subsequent achievement of results. Furthermore, understanding the organizational readiness before introducing a quality improvement intervention in a health-care setting is important. Readiness can potentially influence the degree to which the intervention may become embedded within the health system. From an evaluation perspective, readiness influences the time-to-effect, sometimes rendering the intervention suboptimal for evaluation.

Capacity of the health workforce is one of the key factors influencing health systems readiness in many resource-poor settings. However, capacity building should not be limited to routine clinical and data training, but should also include training in improvement methods. Staff supervision and continuous professional development can increase confidence in clinical skills, but are often lacking in resource-poor settings.^9^ Capacity building can empower health-care providers, patients and communities to ensure that supervisors and decision-makers are accountable and responsive to staff and population needs. For example, increasing capacity to analyse and present data that track health outcomes can be used to hold decision-makers accountable.

Third, strengthened accountability mechanisms promote both efficiency and ownership of services delivery by professionals and communities. The purpose of accountability mechanisms is to ensure that health-care providers get necessary support from other levels of the health system to provide good quality care. One of the mechanisms to achieve accountability is to use evidence within the health system, through the bottom-up generation of quality data and visual presentation of data to decision-makers. This mechanism also includes robust iterative feedback across different levels in the health system. For example, involving patient communities and health-care workers in maternal death surveillance and response systems can improve accountability for maternal deaths and stillbirths.^10^

Fourth, quality improvements should be guided by evidence. However, generating quality data in resource-poor settings has been problematic. Exhaustive data collections, including data from routine health information systems, project monitoring data, improvement data and evaluation data, are rarely translated into meaningful evidence. Although relevant stakeholders within and outside health ministries make efforts to improve data quality from routine monitoring systems, efforts are also needed to harmonize data from the various data sources and optimize the translation of data for building the evidence base. These efforts require significant investments in data quality and data management by stakeholders, including health ministries and implementing agencies.

Sustained improvements in the quality of care delivered by health systems in low-resources settings will only be possible with an expanded evidence base of improvement research in these settings. Methodological thinking and research to support quality improvement work have evolved concurrently. Early work focused on implementation of improvement methods and their comparative effectiveness. The limited or variable effectiveness of some interventions in practice has shifted attention towards understanding the mechanisms by which interventions work, how to optimize their effectiveness and the importance of context.^6^

Fifth, innovative evaluations are important for understanding and advancing the science of quality improvement while also assessing specific intervention efforts. Recent work has demonstrated the value of innovative evaluation models for quality improvement interventions. In these evaluation models traditional scientific definitions of rigour – i.e. unbiased estimates of effect and adequate statistical analysis – are expanded to include use and impact of the improvements interventions. One example is the researcher-in-residence model, which aims to harness the knowledge of all project partners via a researcher embedded within the health-care delivery team who is specifically tasked with understanding the way things operate and how they could be improved.^11^ Another example is cluster randomized-controlled trials with concurrent process and economic evaluations, which allow investigation of specific quality improvement efforts within a larger controlled evaluation of the overall impact of a quality improvement programme.^5^ These models are particularly suited for understanding resource constraints and contextual factors in resource-poor settings and for finding innovative solutions to implementation or evaluation challenges.

While accessible health-care services play a key role in improving health outcomes, greater attention must now be paid to the quality of health care provided, especially in resource-poor settings. The impact and sustainability of quality improvements can be enhanced by adopting a holistic, participatory, accountability-based approach, supported by high-quality innovative evidence generation. An approach resonating well with the participatory, systems thinking that has been advocated for meeting sustainable development goals.^12^ To achieve impact and sustainability, investments are needed to improve data quality, to create an improvement culture and to expand capacity of current and future human resources.
